# Adenosine Receptor mRNA Expression in Frontal Cortical Neurons in Schizophrenia

**DOI:** 10.3390/cells13010032

**Published:** 2023-12-22

**Authors:** Smita Sahay, Emily A. Devine, Robert E. McCullumsmith, Sinead M. O’Donovan

**Affiliations:** 1 Department of Neurosciences, University of Toledo College of Medicine and Life Sciences, Toledo, OH 43614, USA; smita.sahay@rockets.utoledo.edu (S.S.); robert.mccullumsmith@utoledo.edu (R.E.M.); 2 Department of Pharmacology and Systems Physiology, University of Cincinnati College of Medicine, Cincinnati, OH 45267, USA; devineea@mail.uc.edu; 3 Neuroscience Institute Promedica, Toledo, OH 43606, USA

**Keywords:** adenosine receptors, transcript expression, neuromodulation, pyramidal neurons, anterior cingulate cortex, schizophrenia

## Abstract

Schizophrenia is a devastating neuropsychiatric disorder associated with the dysregulation of glutamate and dopamine neurotransmitter systems. The adenosine system is an important neuroregulatory system in the brain that modulates glutamate and dopamine signaling via the ubiquitously expressed adenosine receptors; however, adenosine A_1_ and A_2A_ receptor (A_1_R and A_2A_R) mRNA expression is poorly understood in specific cell subtypes in the frontal cortical brain regions implicated in this disorder. In this study, we assayed A_1_R and A_2A_R mRNA expression via qPCR in enriched populations of pyramidal neurons, which were isolated from postmortem anterior cingulate cortex (ACC) tissue from schizophrenia (*n* = 20) and control (*n* = 20) subjects using laser microdissection (LMD). A_1_R expression was significantly increased in female schizophrenia subjects compared to female control subjects (t_(13)_ = −4.008, *p* = 0.001). A_1_R expression was also significantly decreased in female control subjects compared to male control subjects, suggesting sex differences in basal A_1_R expression (t_(17)_ = 2.137, *p* = 0.047). A significant, positive association was found between dementia severity (clinical dementia rating (CDR) scores) and A_2A_R mRNA expression (Spearman’s r = 0.424, *p* = 0.009). A_2A_R mRNA expression was significantly increased in unmedicated schizophrenia subjects, suggesting that A_2A_R expression may be normalized by chronic antipsychotic treatment (F_(1,14)_ = 9.259, *p* = 0.009). Together, these results provide novel insights into the neuronal expression of adenosine receptors in the ACC in schizophrenia and suggest that receptor expression changes may be sex-dependent and associated with cognitive decline in these subjects.

## 1. Introduction

Schizophrenia is a complex neuropsychiatric illness that affects approximately 0.6% of the U.S. population and is characterized by a range of symptoms broadly classified into positive, negative, and cognitive symptoms [1,2]. Positive symptoms such as hallucinations, delusions, and psychosis are associated with an overactivity of the dopaminergic system [3]. Thus, dopamine D_2_ receptor (D_2_R) antagonism is the mechanism of action for most currently available antipsychotic medications [4,5]. Conversely, negative symptoms such as apathy, social isolation, and anhedonia, as well as cognitive symptoms, like memory impairments and problem-solving, are often associated with dysfunction of the glutamatergic system [6]. The adenosine hypothesis of schizophrenia plays a crucial role in integrating the dopamine and glutamate hypotheses of this disorder, proposing that the dysregulation of these systems is the result of a hypo-adenosinergic state [7].

Adenosine is a purine ribonucleoside involved in numerous physiological processes, including immune modulation, sleep homeostasis, and neuromodulation. As a neuromodulator, adenosine exerts its influence on dopamine and glutamate neurotransmission via a complex system of receptors, enzymes, and transporters found throughout the brain on neurons and glial cells [8]. The central effects of adenosine at physiological concentrations are primarily mediated by the activation of two high-affinity G protein-coupled adenosine receptors: the inhibitory A_1_ receptor (A_1_R) and the excitatory A_2A_ receptor (A_2A_R) [7]. These receptors play a pivotal role in fine-tuning synaptic transmission and serve as key regulators of neurotransmitter release [9].

Of particular interest in schizophrenia are the A_2A_Rs that are predominantly expressed at post-synaptic neurons in the striatum and hippocampus, as well as at intermediate levels in the frontal cortex [7,10,11]. Activation of A_2A_Rs promotes neural excitability, synaptic plasticity, and long-term potentiation, which are widely believed to underlie forms of learning and memory [12]. A_2A_Rs dimerize with D_2_Rs [13], forming heterodimers that facilitate A_2A_R regulation of dopamine function, a phenomenon known to occur in the striatum but that is less established in the frontal cortex brain regions [14,15]. Specifically, A_2A_Rs enhance the inhibitory effects of D_2_Rs, which have been posited to modulate the degree of psychosis and motor hyperactivity observed in schizophrenia [16]. Increasing the expression of A_2A_R-D_2_R heterodimers [17], or targeting A_2A_Rs directly [18], both hold novel therapeutic potential for the treatment of schizophrenia.

Inhibitory A_1_Rs have a widespread distribution in the brain, including in the frontal cortex [7,10,11]. The activation of A_1_Rs inhibits the release of dopamine and glutamate by post-synaptic hyperpolarization [19,20,21]. Dysregulation of this process leads to impairments in memory, problem-solving, and other cognitive processes, all of which are observed in schizophrenia [22,23]. Increased dopamine turnover has also been well documented in schizophrenia, a finding compatible with adenosine deficiency, as adenosine binding to A_1_Rs inhibits dopamine release via A_1_R-dopamine D_1_ receptor (D_1_R) heterodimer formation [8,24,25]. Adenosine deficiency in schizophrenia also potentiates amphetamine-induced locomotion and dopamine release, as suggested by the effects of A_1_R antagonists [26]. Animal model studies of schizophrenia have shown that, although the hypofunction of glutamate and/or its N-methyl-D-aspartate receptor (NMDAR) leads to cognitive decline, behavioral dysfunction, and psychosis, NMDAR co-agonists (e.g., D-serine and glycine) show efficacy in improving cognition [27], and adenosine receptor agonists show efficacy in improving behavior and psychotic symptoms [8].

Postmortem brain tissue studies have reported dysregulated A_2A_R mRNA and protein levels in the hippocampus [28,29] and striatum [29,30,31] of schizophrenia subjects. A pronounced reduction in the formation of A_2A_R-D_2_R heterodimers was reported in the caudate nucleus in schizophrenia, although individual receptor expression was increased in the same study [17]. Fewer postmortem studies have focused on the A_1_R and its cellular expression, with no significant changes in mRNA expression found in the striatum in schizophrenia [31], but reduced A_1_R mRNA expression in pyramidal neurons in the dorsolateral prefrontal cortex (DLPFC) in schizophrenia [32]. Together, these findings support the theory of adenosine receptor dysregulation, which profoundly impacts neuromodulatory function and is implicated in schizophrenia’s pathophysiology [22,33]; yet, little is known about their mRNA expression in specific frontal cortical cell types in patients diagnosed with this disorder.

In the present study, we assayed the transcript expression of A_1_Rs and A_2A_Rs in enriched populations of pyramidal neurons in the anterior cingulate cortex (ACC). We maintained a focus on pyramidal neurons due to the convergence of genetic risk in schizophrenia on glutamatergic synapses [34,35]. Dysregulation of the ACC, which is involved in cognition, executive functioning, emotional processing, and social behavior [36,37], is implicated in schizophrenia, and, as previously stated, evidence suggests the presence of adenosine receptors here [38,39]. Our findings provide a more robust understanding of adenosine receptor gene expression in the brain in schizophrenia and a broader appreciation of the adenosine hypothesis of this disorder.

## 2. Materials and Methods

### 2.1. Subjects

Postmortem human brain tissue was sourced from the Bronx–Mount Sinai NIH Brain and Tissue Repository, with the appropriate consent obtained from the next of kin under IRB-approved protocols. The specimens, comprising individuals without psychiatric illness (*n* = 20) and those diagnosed with schizophrenia (*n* = 20) from the anterior cingulate cortex (ACC), were matched for sex, age, postmortem interval (PMI), and pH (Table 1). Following dissection, brain tissues were promptly frozen at −80 °C until required for analysis. Diagnoses were independently established by two psychiatrists through a comprehensive review of medical records, autopsy reports, and family interviews using the Structured Clinical Interview for the Diagnostic and Statistical Manual of Mental Disorders, Fourth Edition (DSM-IV). Antipsychotic drug(s) (APD) status was categorized as “on” for subjects taking medication in the last six weeks of life. Detailed demographic information is provided in Supplementary Table S1.

### 2.2. Laser Microdissection (LMD)

Enriched populations of ACC pyramidal cells were identified by utilizing Nissl staining techniques to conduct cell-level investigations of adenosine receptor mRNA expression. LMD was performed using the Leica Laser Microdissection 6 instrument (Leica Microsystems, Wood Dale, IL, USA) to precisely cut stained pyramidal neurons from the superficial (layers II–III) and deep (layers V–VI) grey matter of the ACC. The LMD procedures adhered to validated methods, as previously outlined [32,40,41,42]. Briefly, frozen tissue sections were thawed at room temperature and rehydrated with distilled H20, followed by rapid Nissl staining using an RNAse-free cresyl-violet solution (1% cresyl violet, 1% glacial acetic acid, pH 4.0) (FD NeuroTechnologies, Columbia, MD, USA). After ethanol washes and histoclear treatment, enriched pyramidal neuron populations (500 per subject) were identified based on morphology and excised from the ACC grey matter via LMD. Microdissection was conducted under a 40X objective lens with the following laser settings: power: 24–25, aperture: 4–5, and speed: 8. The dissected cells were collected into the caps of separate 0.5 mL tubes (Axygen, Union City, CA, USA) for each subject, incubated with 30 μL of PicoPure RNA extraction buffer (Applied Biosystems, Foster City, CA, USA) for 32 min at 42 °C, centrifuged for 2 min at 400× *g*, and stored at −80 °C until further processing.

Although these samples were highly enriched [41,42,43], they may have contained neuropil, processes, or other small cells, including interneurons; however, we have published this method extensively and have shown successful enrichment of neurons (see Supplementary Methods). Overall, LMD is a reliable and feasible tool for capturing specific cell types from postmortem human brain tissue [44].

### 2.3. RNA Isolation, Reverse Transcription, and Complementary DNA (cDNA) Pre-Amplification

RNA isolation from enriched pyramidal cell populations was executed using the PicoPure RNA isolation kit, following the prescribed guidelines from the manufacturer (Molecular Devices, Sunnyvale, CA, USA). For cDNA synthesis, the High-Capacity cDNA Reverse Transcription Kit (Applied Biosystems, Foster City, CA, USA) was employed, utilizing 10 μL of total RNA. TaqMan primers for A_1_R (ADORA1); A_2A_R (ADORA2A); and housekeeping genes cyclophilin A (PPIA), beta actin (ACTB), beta2-microglobulin (B2M), and glyceraldehyde-3-phosphate dehydrogenase (GAPDH) were pooled, diluted with RNAse/DNase-free water to a final concentration of 0.2X, and combined with FastStart Universal Mastermix (Roche Life Sciences, IN, USA) and cDNA for the pre-amplification polymerase chain reaction (PCR). The PCR cycles comprised 1 cycle of denaturing at 95 °C for 10 min, followed by 14 cycles of denaturing at 95 °C for 14 s, and annealing at 60 °C for 4 min. After pre-amplification, samples were diluted at a 1:5 ratio with RNase-free water and preserved at −20 °C until they were used for real-time quantitative PCR (qPCR) assays.

### 2.4. Quantitative Real-Time Polymerase Chain Reaction (RT-qPCR)

The qPCR reactions were executed for each subject in duplicate using 96-well optical reaction plates (Life Technologies, Carlsbad, CA, USA) on an Applied Biosystems detection system (ABI SteponePlus, Life Technologies, USA). Each reaction comprised 3 µL of pre-amplified cDNA in a 20 µL reaction containing 10 µL of mastermix and a 1X dilution of each primer (Applied Biosystems, Life Technologies, USA). The primers utilized are detailed in Supplementary Table S2. Reaction conditions involved an initial ramp time of 10 min at 95 °C, followed by 40 cycles of 15 s at 95 °C, and 1 min at the annealing temperature of 60 °C. Negative controls for the assay encompassed the omission of cDNA (non-template control) or the generation of cDNA with reverse transcriptase (RT) excluded from the reaction (no RT control). Relative concentrations of ADORA1 and ADORA2A, the transcripts of interest, were computed relative to a standard curve established with cDNA dilutions from a pooled sample of all the subjects. Transcript values of interest were normalized to the geometric mean of B2M, ACTB, GAPDH, and PPIA, reference genes with unaltered expression in both the control and schizophrenia groups (Student’s *t*-test, *p* > 0.05), for all subjects in the study.

### 2.5. Data Analysis

All datasets underwent testing for normal distribution (D’Agostino and Pearson omnibus normality test) and homogeneity of variance (F-test). Log transformation was applied to the data, and outliers were identified using the ROUT method (Q = 1%). Multiple regression analyses were conducted to ascertain associations between dependent measures (ADORA1 and ADORA2A transcript expression) and age and postmortem interval (PMI). Analysis of covariance (ANCOVA) was employed in cases where significant associations were detected. In the absence of significant associations, data were subjected to an unpaired two-tailed Student’s *t*-test. Bonferroni’s post hoc analyses were subsequently conducted for all significant findings to address the issue of multiple comparisons. Spearman’s correlation analysis was conducted to quantify the association between transcript expression and clinical dementia rating (CDR) scores. CDR scores were categorized into three groups: subjects with no dementia symptoms (Bin 1, CDR score 0), subjects with very mild to mild dementia symptoms (Bin 2, CDR score 0.5–1), and subjects with moderate to severe dementia symptoms (Bin 3, CDR score 2–3). α = 0.05 for all statistical tests. Data were analyzed using Statistica 13.0 (Statsoft, Tulsa, OK, USA) and Graphpad Prism 7.04 (GraphPad Software, La Jolla, CA, USA, www.graphpad.com).

## 3. Results

We measured and compared the mRNA expression levels of the adenosine A_1_Rs and A_2A_Rs in an enriched population of ACC pyramidal neurons between schizophrenia and sex- and age- matched non-psychiatrically-ill control subjects.

### 3.1. Adenosine Receptor Transcript Expression

**Figure 1:**
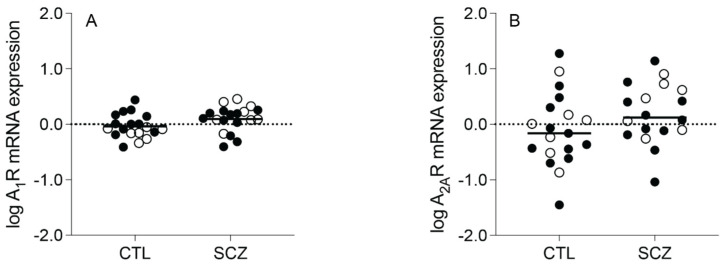
Adenosine A_1_ receptor (A_1_R) and A_2A_ receptor (A_2A_R) mRNA expression in an enriched population of anterior cingulate cortex (ACC) pyramidal neurons in control (CTL) vs. schizophrenia (SCZ) subjects. (**A**) A_1_R and (**B**) A_2A_R mRNA expression was not significantly different between SCZ and CTL subjects. *n* = 18–19/group. Open circles indicate female subjects and closed circles indicate male subjects. Data are presented as means.

A_1_R mRNA expression (t_(36)_ = −1.853, *p* = 0.072) and A_2A_R mRNA expression (F_(1,34)_ = 1.632, *p* = 0.210, after controlling for the effect of PMI) were not significantly different between the schizophrenia and control subjects (Figure 1A,B).

**Figure 2:**
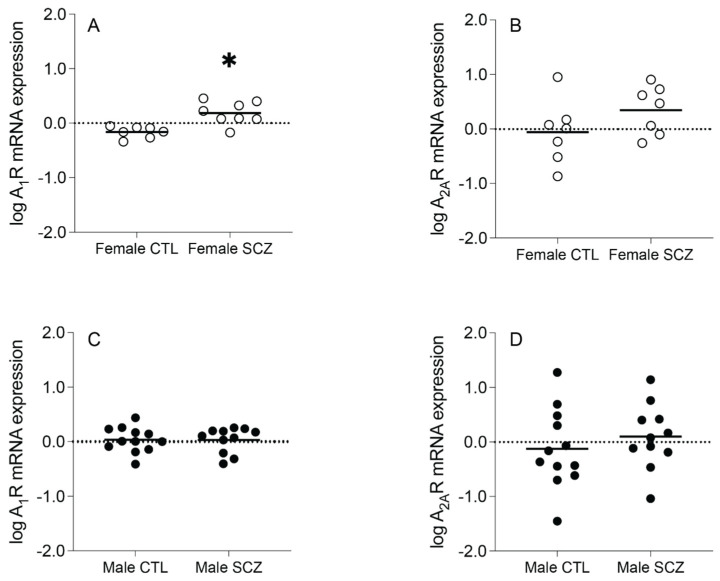
Adenosine A_1_ receptor (A_1_R) and A_2A_ receptor (A_2A_R) mRNA expression in an enriched population of anterior cingulate cortex (ACC) pyramidal neurons in female control (CTL) vs. schizophrenia (SCZ) subjects and male CTL vs. SCZ subjects. (**A**) A_1_R mRNA expression was significantly increased in female SCZ subjects compared to female CTL subjects. (**B**) A_2A_R mRNA expression was not significantly different between female SCZ subjects and female CTL subjects. (**C**) A_1_R mRNA expression and (**D**) A_2A_R mRNA expression were not significantly different between male SCZ subjects and male CTL subjects. *n* = 7–12/group. Open circles indicate female subjects and closed circles indicate male subjects. Data are presented as means. * *p* < 0.05.

A_1_R mRNA expression (t_(13)_ = −4.008, *p* = 0.001), but not A_2A_R mRNA expression (t_(12)_ = −1.466, *p* = 0.168), was significantly increased in female schizophrenia subjects compared to female control subjects (Figure 2A,B). A_1_R mRNA expression (t_(21)_ = 0.049, *p* = 0.962) and A_2A_R mRNA expression (F_(1,20)_ = 1.533, *p* = 0.230, after controlling for the effect of PMI) were not significantly different between male schizophrenia subjects and male control subjects (Figure 2C,D).

**Figure 3:**
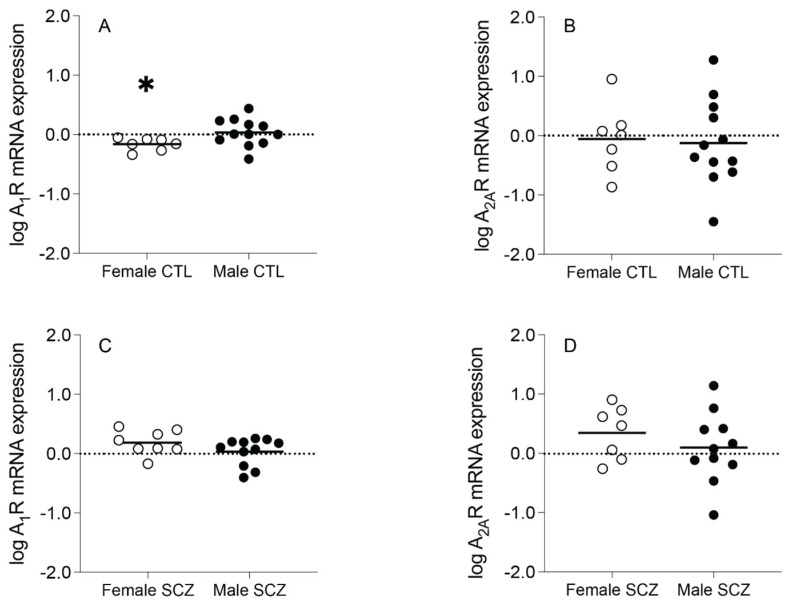
Adenosine A_1_ receptor (A_1_R) and A_2A_ receptor (A_2A_R) mRNA expression in an enriched population of anterior cingulate cortex (ACC) pyramidal neurons in control (CTL) female vs. male subjects and female vs. male schizophrenia (SCZ) subjects. (**A**) A_1_R mRNA expression was significantly decreased in female CTL subjects compared to male CTL subjects. (**B**) A_2A_R mRNA expression was not significantly different between female CTL subjects and male CTL subjects. (**C**) A_1_R mRNA expression and (**D**) A_2A_R mRNA expression were not significantly different between female SCZ subjects and male SCZ subjects. *n* = 7–12/group. Open circles indicate female subjects and closed circles indicate male subjects. Data are presented as means. * *p* < 0.05.

A_1_R mRNA expression (t_(17)_ = 2.137, *p* = 0.047), but not A_2A_R mRNA expression (t_(17)_ = −0.206, *p* = 0.840), was significantly decreased in female control subjects compared to male control subjects (Figure 3A,B). A_1_R mRNA expression (t_(17)_ = 1.495, *p* = 0.153) and A_2A_R mRNA expression (t_(16)_ = 0.948, *p* = 0.357) were not significantly different between female schizophrenia and male schizophrenia subjects (Figure 3C,D).

### 3.2. Adenosine Receptor Transcript Expression and CDR Bins

**Figure 4:**
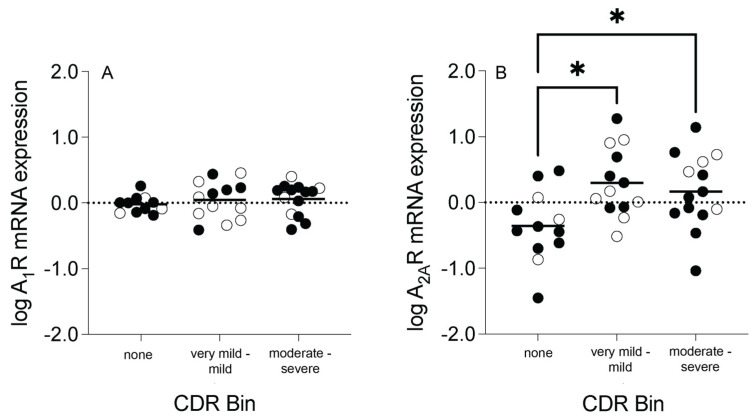
Adenosine A_1_ receptor (A_1_R) and A_2A_ receptor (A_2A_R) mRNA expression in an enriched population of anterior cingulate cortex (ACC) pyramidal neurons in control (CTL) and schizophrenia (SCZ) subjects, binned according to clinical dementia rating (CDR) score. (**A**) A_1_R mRNA expression was not significantly different across CDR bins. (**B**) A_2A_R mRNA expression was significantly increased in subjects in the “very mild–mild” and “moderate–severe” bins compared to subjects with no dementia symptoms (“none” bin). *n* = 11–14/group. Open circles indicate female subjects and closed circles indicate male subjects. Data are presented as means. * *p* < 0.05.

CDR scores were binned into three groups: subjects with no dementia symptoms (bin 1, CDR score 0), subjects with very mild to mild dementia symptoms (bin 2, CDR score 0.5–1), and subjects with moderate to severe dementia symptoms (bin 3, CDR score 2–3). A_2A_R mRNA expression (F_(2,32)_ = 5.904, *p* = 0.007, after controlling for the effect of PMI) was significantly different among the CDR bins. After post hoc analyses, significant increases were observed between bins 1 and 2 (*p* = 0.006) and bins 1 and 3 (*p* = 0.012), but not between bins 2 and 3 (*p* = 1.000) (Figure 4B). A_1_R mRNA expression (F_(2,35)_ = 1.263, *p* = 0.295) was not significantly different among the CDR bins (Figure 4A). No significant sex differences were observed in A_1_R or A_2A_R mRNA expression among the CDR bins (*p* > 0.05, Supplementary Figure S1).

### 3.3. Correlation Analysis between Adenosine Receptor Transcript Expression and CDR Scores

**Figure 5:**
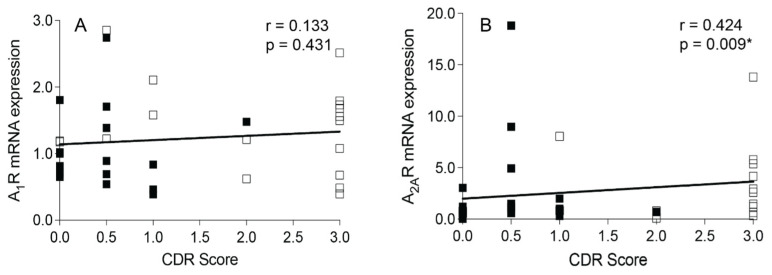
Spearman’s correlation analysis between adenosine A_1_ receptor (A_1_R) and A_2A_ receptor (A_2A_R) mRNA expression and clinical dementia rating (CDR) scores in control (CTL) and schizophrenia (SCZ) subjects. (**A**) No significant associations were observed between A_1_R mRNA expression and CDR scores. (**B**) A significant, positive association was observed between A_2A_R mRNA expression and CDR scores. CDR scores: 0 = no dementia, 0.5–1 = very mild to mild dementia, 2–3 = moderate to severe dementia. *n* = 37. Open squares indicate SCZ subjects and closed squares indicate CTL subjects. * *p* < 0.05.

A significant association was observed between A_2A_R mRNA expression and CDR scores (Spearman’s r = 0.424, *p* = 0.009, Figure 5B), but not between A_1_R mRNA expression and CDR scores (Spearman’s r = 0.133, *p* = 0.431, Figure 5A). No significant associations were observed between adenosine receptor transcript expression and CDR scores in females or males (*p* > 0.05, Supplementary Figure S2).

### 3.4. Effect of Antipsychotic Medications on Adenosine Receptor Transcript Expression

Adenosine receptor expression was analyzed in schizophrenia subjects who were “on” compared to “off” medication at the time of death. Subjects were considered “off” medication if they were not taking antipsychotics for at least six weeks prior to death. Subjects whose medication status was unknown were excluded from the analysis (*n* = 2).

**Figure 6:**
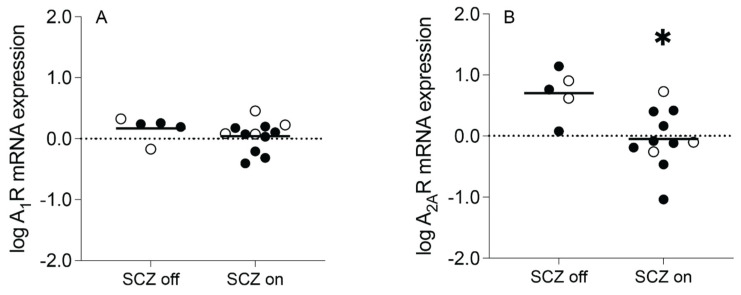
Adenosine A_1_ receptor (A_1_R) and A_2A_ receptor (A_2A_R) expression in an enriched population of anterior cingulate cortex (ACC) pyramidal neurons in schizophrenia (SCZ) subjects off vs. on antipsychotic medication. (**A**) A_1_R mRNA expression was not significantly different between SCZ subjects on antipsychotic medication and SCZ subjects off antipsychotic medication. (**B**) A_2A_R mRNA expression was significantly decreased in SCZ subjects on antipsychotic medication compared to SCZ subjects off antipsychotic medication. *n* = 5–12/group. Open circles indicate female subjects and closed circles indicate male subjects. Data are presented as means. * *p* < 0.05.

A_2A_R mRNA expression (t_(14)_ = −3.043, *p* = 0.009), but not A_1_R mRNA expression (t_(15)_ = −1.031, *p* = 0.319), was significantly decreased in schizophrenia subjects “on” medication compared to schizophrenia subjects “off” medication. This finding was driven by male (t_(9)_ = −2.321, *p* = 0.045), and not female (t_(3)_ = −1.558, *p* = 0.217), schizophrenia subjects “on” medication (Figure 6A,B). A_2A_R mRNA expression (t_(22)_ = −2.578, *p* = 0.017), but not A_1_R mRNA expression (t_(22)_ = −1.943, *p* = 0.065), was also significantly increased in schizophrenia subjects “off” medication compared to non-psychiatrically-ill control subjects, suggesting a disease-related increase in A_2A_R mRNA expression that may be normalized by antipsychotic treatment (Supplementary Figure S3C,D). No significant differences were observed in A_1_R or A_2A_R mRNA expression in schizophrenia subjects “on” medication compared to non-psychiatrically ill control subjects (*p* > 0.05, Supplementary Figure S3A,B); however, A_1_R mRNA expression was significantly increased in female (t_(9)_ = −4.444, *p* = 0.002), but not male (t_(18)_ = 0.748, *p* = 0.464), schizophrenia subjects “on” medication compared to control subjects.

## 4. Discussion

Schizophrenia remains a challenging neuropsychiatric disorder to treat, with considerable impacts on the lives of affected individuals and their families. In recent years, attention has turned to the adenosine system and its potential neuromodulatory role in the pathophysiology of schizophrenia [8,15,32,45], as well as the potential for adenosine receptors specifically as therapeutic substrates for this disorder [45,46]. The adenosine receptors are expressed throughout the human brain [47], with most studies focusing on changes in receptor expression and function in the striatum and striatal–dopamine neurons in schizophrenia [7,10,11,29,30,31]. Much less is known about adenosine receptor expression in the neurons of other frontal cortical brain regions, like the ACC, that are implicated in schizophrenia. This study, focusing on assaying adenosine receptor mRNA expression in enriched populations of pyramidal neurons in the ACC, provides insight into the potential role of the adenosine system in this illness and suggests that receptor mRNA expression changes may be sex-dependent and associated with cognitive decline in the assayed subjects.

Previous studies have established sex-related differences in the clinical presentation and course of schizophrenia, including the age of onset and symptom severity, with an earlier age of onset prevalent in males [48,49,50]. Our study found increased A_1_R mRNA levels in an enriched population of ACC pyramidal neurons in female schizophrenia subjects compared to non-psychiatrically-ill female control subjects, supporting the broader notion of heterogeneity in this illness at the transcript expression level. An increase in A_1_R expression may indicate an inhibition of glutamate release, leading to decreased excitatory signaling. A_1_R antagonists may, therefore, be expected to potentiate glutamatergic activity, including NMDAR-dependent activation, leading to cognitive improvement: a theory supported by the pro-cognitive effects of the non-specific adenosine receptor antagonist caffeine [45,51,52]. Notably, however, alterations in mRNA do not necessarily correlate with corresponding shifts in protein levels, and further studies investigating adenosine receptor protein abundance and activity levels at the cell-subtype level are crucial to understanding the downstream implications of this finding.

On the other hand, A_1_R genetic deletion has resulted in animals displaying aggression and anxiety with no effects on their spatial or working memory [53]. A_1_R agonists have shown efficacy against behavioral and neurophysiological (e.g., prepulse inhibition) effects induced by NMDAR antagonists in animal models of schizophrenia [54,55,56]. In amphetamine and MK-801 (dizocilpine) rodent models used to mimic schizophrenia endophenotypes, selective A_1_R agonists reduced hyperlocomotion, thereby acting as effective antipsychotics [57]. Conversely A_1_R agonists decreased overall learning and memory performance in non-pathological [58,59] and memory impairment animal models [60].

Similarly, several studies have reported conflicting evidence on the therapeutic benefits of A_2A_R agonists and antagonists in schizophrenia. Like A_1_R agonists, A_2A_R agonists generally attenuate prepulse inhibition deficits [18,61,62] and effectively antagonize the motor effects induced by psychostimulant drugs [63,64]; yet, they consistently result in memory deficits in preclinical models [65,66,67]. Adenosine also acts synergistically with NMDARs in A_2A_R-enriched brain regions such as the striatum, which partially explains the efficacy of A_2A_R agonists against the psychostimulant effects of NMDAR antagonists [54,68]. Few studies have reported null effects of antagonizing motor behavior [14] and working memory [69] with A_2A_R agonists, although one study utilizing electrophysiology techniques reported that long-term potentiation (LTP) is prevented by A_2A_R antagonism, suggesting that A_2A_R activation may actually facilitate memory-related processes [70].

A_2A_R antagonism has been reported to enhance memory performance in non-pathological animal models and to ameliorate the loss of memory functions [67,71]. A_2A_Rs increase the expression of the calcium-permeable GluA1 subunit of the α-amino-3-hydroxy-5-methyl-4-isoxazolepropinonic acid (AMPA) receptor through the activation of protein kinase A [72], which, in part, explains the excitatory neuromodulatory effect associated with positive symptoms in schizophrenia and the potential antipsychotic benefit of A_2A_R antagonists.

Interestingly, preclinical and clinical data have reported the benefit of A_1_R activation in neurological conditions such as epilepsy [21], stroke [21], and chronic pain [73], as well as the benefit of A_2A_R hypofunction in stroke [21], chronic pain [74], Parkinson’s [75], and Alzheimer’s [76]. The current antipsychotic therapeutic potential of A_1_R agonists and A_2A_R antagonists in schizophrenia is largely based on preclinical models and theoretical considerations; however, further studies investigating the underlying molecular basis for these considerations such as those examined in the present study, and, of course, clinical evidence, have yet to be established [8,22].

The extensive studies reviewed above implicate the dysregulation of A_1_R and A_2A_R in behaviors associated with schizophrenia and highlight the seemingly effective potential of targeting the adenosine receptor system to regulate dopaminergic hyperfunction and NMDAR hypofunction. Additionally, increased mRNA and/or protein levels of A_1_Rs and A_2A_Rs have been reported in the frontal cortex in Pick’s disease [77], Alzheimer’s disease [78], and Creutzfeldt–Jakob disease [79], providing additional support for adenosine receptor dysregulation specifically in frontal cortical brain regions in neurocognitive disorders, as well as for the use of adenosine-based therapeutic strategies [45,80]. In our study, we did not detect significant differences in A_2A_R mRNA expression between schizophrenia and control groups overall or in either sex; however, this may have been due to a relatively smaller sample size, especially in the sex comparisons.

Reports investigating sex differences in the adenosine receptor system utilizing positron emission tomography (PET) have shown increased A_1_R availability in female control subjects compared to male control subjects, with the largest difference in receptor availability observed in the ACC [81,82]. Similarly, we identified a difference in basal A_1_R mRNA expression between female and male control subjects in the ACC, but with lower levels in females. This observation adds to the growing body of evidence highlighting sexual dimorphism in various aspects of the brain, including neurotransmitter systems, receptor mRNA and protein levels, and neural connectivity [35,83].

In contrast, another study investigating the availability of A_2A_Rs utilizing PET showed no significant differences in receptor binding potential between male patients with chronic schizophrenia and matched control subjects [84]. Our study also did not identify a difference in A_2A_R mRNA expression between schizophrenia and matched subjects in either sex. However, as mentioned, these findings may be limited by a relatively small sample size (12 male patients vs. 13 male controls in the PET study, and 7 female patients vs. 7 female controls in our study). A larger cohort of samples inclusive of both genders is warranted in any future postmortem qPCR studies, and is feasible given that many of the well-established brain banks are growing their collections of specimens and increasing the number of high-quality, well-matched samples [85,86].

An additional analysis in our study was performed to assess the relationship between A_1_R and A_2A_R mRNA expression and CDR scores among all subjects. The CDR is a tool to assess cognitive decline and its impact on daily functioning. Lower scores indicate little to no cognitive decline, whereas higher scores indicate the presence of dementia [87]. We observed a significant increase in A_2A_R expression in subjects with very mild to mild dementia (bin 2) and with moderate to severe dementia (bin 3) relative to those with no cognitive decline (bin 1). Furthermore, a positive correlation between A_2A_R expression and CDR scores was evident. These findings raise questions about the role of A_2A_Rs in the progression of cognitive decline. A substantial amount of research suggests that adenosine receptors modulate cognitive function [8,88]. Human and animal studies support that adenosine receptor activity can reverse cognitive impairments in animal models of schizophrenia [89], Alzheimer’s disease [90], Parkinson’s disease [91], and Huntington’s disease [92,93]. Epidemiological evidence indicates that routine human consumption of caffeine, a nonselective adenosine receptor antagonist and the most widely consumed psychoactive drug, is associated with reduced cognitive decline in aging and Alzheimer’s patients, as well as a reduced risk of developing Parkinson’s [94,95]. Clinical trials have shown the motor benefits of A_2A_R antagonists in Parkinson’s patients with high safety profiles [91,94]; however, additional studies are needed to understand the underlying mechanism of adenosine-receptor-mediated control of cognition under normal and disease conditions across neurocognitive disorders, including schizophrenia. Our findings support the idea that A_2A_Rs may perhaps serve as a biomarker of cognitive deficits in schizophrenia, and future investigations are necessary to understand whether modulating A_2A_R activity may offer an efficacious therapeutic avenue for addressing cognitive decline.

To determine whether changes in adenosine receptor mRNA expression are secondary to antipsychotic treatment or inherent to the disease process itself [7], we investigated the impact of antipsychotic medications on A_1_R and A_2A_R expression in schizophrenia subjects. We found that A_2A_R mRNA expression was reduced in schizophrenia subjects treated with antipsychotics compared to off-antipsychotic schizophrenia subjects, but A_2A_R mRNA expression was increased in the off-medication subjects compared to the control subjects. Collectively, this suggests that A_2A_R expression is elevated in schizophrenia, an effect that may be normalized by chronic antipsychotic treatment. A previous study measuring the mRNA expression of significantly altered adenosinergic targets in rats treated for nine months with haloperidol decanoate found a significant decrease in A_1_R mRNA expression in an enriched population of DLPFC pyramidal neurons in the haloperidol-treated rats compared to controls [32]. Another postmortem study of prefrontal cortex tissue revealed no significant differences in A_2A_R mRNA or protein levels between antipsychotic-treated schizophrenia subjects and drug-free schizophrenia or healthy control subjects [96]. As our study assayed A_2A_R mRNA expression in enriched pyramidal neuron populations captured from the ACC, this may suggest brain-cell-subtype-specific changes in adenosine receptor expression that may not be captured at the brain region level. Others have reported a positive correlation between antipsychotic dosage and A_2A_R density [29,30] in striatal postmortem brain tissue and increased A_2A_R mRNA expression in platelets after six weeks of antipsychotic treatment that could not be replicated in antipsychotic-free patients [97]. These reports suggest that adenosine receptor expression may be altered by antipsychotic medication; however, we found that treatment may mask disease-related increases in neuronal A_2A_R mRNA expression in the ACC.

A key point to note regarding the medication analysis is that information regarding the use of antipsychotic or over-the-counter medications (e.g., medication name, class of medication, duration of use) by all subjects prior to death may have had an impact on A_1_R and/or A_2A_R mRNA expression; however, this information was not readily available for use in this study. Our analysis also included a relatively small number of off-antipsychotic medication subjects, which is not surprising given the severity of this disorder; however, these findings should be confirmed in a larger study that also considers the effect of different classes of antipsychotics and non-antipsychotic medications, when possible, to determine the effects of medications on adenosine receptor mRNA expression more precisely.

An important consideration in interpreting the above findings involves the gene expression levels of the enzymes related to the synthesis, transport, and degradation of the endogenous ligand adenosine, as well as their impact on adenosine receptor levels. In neurons, adenosine is directly released from cells via bidirectional equilibrative nucleoside transporters (ENTs) [98]. Specifically, the ENT1 transporter equilibrates adenosine concentration across membranes [20]. A previous study investigating ENT1 mRNA in schizophrenia reported reduced levels in DLPFC pyramidal neurons [32]. Consistently, others have reported decreased ENT1 protein levels in the superior temporal gyrus of elderly schizophrenia subjects; however, no change was reported in ENT1 protein levels in the ACC [99]. Another report showed ENT1-dependent release of adenosine in response to firing neurons, resulting in increased inhibitory A_1_R activation [100]. They further reported this was independent of 5′-nucleotidase (5′NT), an enzyme involved in the extracellular catabolism of ATP to adenosine. This suggests that neuronal ENT1 expression may be correlated with decreased adenosine levels both intra- and extra- cellularly, a finding that contributes to the theory of cell-subtype-specific gene expression dysfunction of adenosinergic targets in schizophrenia. Another enzyme associated with the catabolism of extracellular adenosine to inosine is adenosine deaminase (ADA) [101]. The same study that reported reduced ENT1 mRNA in DLPFC pyramidal neurons in schizophrenia found increased levels of the irreversible enzyme ADA [32], suggesting lower levels of adenosine in schizophrenia compared to control subjects. Although ENT, 5′NT, and ADA were not targets of interest in the present study, further studies will aim to identify the expression levels of these neuronal enzymes in the ACC, as well as their correlations with A_1_Rs and A_2A_Rs, to understand whether the expression levels of adenosine receptors are altered solely due to a disease effect or because of altered adenosine levels in the cerebral spinal fluid.

Overall, our study sought to assess the distinct transcriptional profile of the high-affinity adenosine receptors in ACC pyramidal neurons in schizophrenia to understand the early molecular events underlying the pathophysiology of this disorder. As stated, changes in mRNA levels do not always indicate a similar change in protein levels. Although we have discussed the observed changes in A_1_R and A_2A_R mRNA expression in enriched populations of frontal cortical neurons in this disorder, future postmortem studies considering protein abundance and activity of adenosinergic targets using larger sample sizes and brain tissue from other implicated brain regions are warranted to gain a broader biochemical understanding of schizophrenia’s pathology. Additionally, as all analyses performed herein were retrospective, an inherent limitation of postmortem studies. Preclinical experimental models are crucial to testing mechanistic hypotheses, further explaining postmortem observations, and determining the functional consequences of adenosine receptor dysregulation in schizophrenia.

## 5. Conclusions

In conclusion, our study focuses on the transcript expression of high-affinity A_1_Rs and A_2A_Rs in enriched populations of ACC pyramidal neurons in schizophrenia. We identified sex-specific alterations in A_1_R mRNA expression as well as sexual dimorphism in basal A_1_R mRNA levels, highlighting the importance of considering sex as a factor when investigating the molecular basis of this disorder. We observed that subjects with more severe cognitive decline, based on higher CDR scores, had significantly higher levels of A_2A_R mRNA levels regardless of sex, providing further support for the role of adenosine receptors in the modulation of cognitive function as well as for their potential to serve as therapeutic targets for addressing cognitive deficits. Lastly, our medication analysis indicated that antipsychotic medications may normalize A_2A_R mRNA levels in the ACC. These findings collectively contribute to a deeper understanding of the adenosine receptor system’s role in schizophrenia’s pathophysiology, paving the way for more targeted, sex-specific, and evidence-based therapeutic approaches in the future.

## Figures and Tables

**Table 1:** Subject demographics. Data are presented as mean ± standard deviation. Data ranges are in parenthesis. Abbreviations: *N*, number of subjects; F, female; M, male; PMI, postmortem interval; CDR, clinical dementia rating; Med, medication; N/A, not applicable.

	*N*	Sex	Age	PMI (hours)	pH	CDR	Medications
Schizophrenia	20	9 F/11 M	75 ± 8 (61–90)	13.1 ± 5.8 (5.8–24)	6.3 ± 0.2 (5.85–6.74)	2.3 ± 2 (0–3)	F: 4 On/3 Off/2 Unknown M: 8 On/3 Off/0 Unknown
Control	20	7 F/13 M	78 ± 7 (64–86)	12.4 ± 7.5 (3.3–24)	6.6 ± 0.4 (6.04–7.27)	0.4 ± 0.5 (0–3)	N/A

## Data Availability

Data are contained within the article and Supplementary Materials.

## Supplementary Materials

The following supporting information can be downloaded at: https://www.mdpi.com/article/10.3390/cells13010032/s1, Supplementary Methods: Quality control studies demonstrating enrichment of pyramidal neurons in postmortem tissue; Table S1: Demographics of individual subjects; Table S2: TaqMan primers used in study; Figure S1: A_1_R and A_2A_R mRNA expression in an enriched population of ACC pyramidal neurons in CTL and SCZ subjects, binned according to CDR score in female and male subjects; Figure S2: Spearman’s correlation analysis between A_1_R and A_2A_R mRNA expression and CDR scores in female and male subjects; Figure S3: A_1_R and A_2A_R mRNA expression in an enriched population of ACC pyramidal neurons in SCZ subjects on or off antipsychotic medication vs. CTL subjects. Refs. [32,41,42] are cited in Supplementary Files.
