# Supplementary material for: Adenosine Receptor mRNA Expression in Frontal Cortical Neurons in Schizophrenia
Source: Cells. 2023 Dec 22;13(1):32. doi: 10.3390/cells13010032 (PMC10778287; doi:10.3390/cells13010032)
Supplement: Supplementary file 1 [file cells-13-00032-s001.zip › cells-2722181-supplementary.pdf]

<u>Supplementary Material</u>.

Contents.

1. **Supplementary Methods** Page 2
2. **Supplementary Tables & Captions** Page 3
3. **Supplementary Figures & Captions** Page 5
4. **Supplementary References** Page 8

Supplementary Methods.

Quality control studies demonstrating enrichment of pyramidal neurons in postmortem tissue.

The application of the laser microdissection (LMD) methodology in isolating human postmortem tissue is relatively novel. We have previously published extensive quality control studies for LMD-qPCR in human brain [1] in the supplemental materials of [2, 3], demonstrating that the distinctive size and shape of pyramidal cells allows for accurate capture of these cells by Nissl staining and morphological identification.

Briefly, we utilized human postmortem tissue from the anterior cingulate cortex (ACC). We consistently harvested 500 large cells with pyramidal-neuron morphology and 500 small cells from the same tissue section in about three hours. Small cells with a circular, punctuate nucleus were assumed to be a mixture of glia and interneurons. These enriched cell populations were assayed using qPCR with vesicular glutamate transporter 1 (VGLUT1) and neuron specific enolase (NSE), markers for pyramidal neurons.

The large pyramidal neurons in the deep layer of the cortical matter were readily identifiable. The large cells were enriched for VGLUT1, while the large and small cells were both equally enriched for NSE, consistent with the presence of neurons in both large and small cell preparations. Overall, these quality control studies showed that enriched populations of neurons may be successfully harvested based on morphological identification.

Supplementary Tables & Table Captions.

SUBJECT #	AGE	RACE	SEX	PH	PMI	CDR	MED STATUS	DIAGNOSIS
**1**	85	W	M	N/A	5.3	0	N/A	Control
**2**	78	W	M	N/A	8.1	2	N/A	Control
**3**	78	W	F	6.48	4.3	1	N/A	Control
**4**	75	B	M	6.43	5.0	0	N/A	Control
**5**	85	W	F	N/A	5.3	1	N/A	Control
**6**	86	W	F	N/A	11.5	0.5	N/A	Control
**7**	78	W	M	N/A	5.9	0	N/A	Control
**8**	85	N/A	M	N/A	16.2	0	N/A	Control
**9**	70	W	M	6.04	23.8	0	N/A	Control
**10**	64	W	M	6.12	10.4	0	N/A	Control
**11**	85	W	F	7.27	8.0	0	N/A	Control
**12**	68	W	F	6.30	24.0	0	N/A	Control
**13**	71	W	M	N/A	5.6	0	N/A	Control
**14**	75	H	F	N/A	3.3	0.5	N/A	Control
**15**	84	W	M	6.62	20.9	1	N/A	Control
**16**	71	W	M	7.09	21.7	0	N/A	Control
**17**	73	W	M	6.94	21.1	0.5	N/A	Control
**18**	86	W	F	N/A	10.2	0.5	N/A	Control
**19**	71	W	M	N/A	21.4	0.5	N/A	Control
**20**	84	H	M	N/A	16.8	0.5	N/A	Control
**21**	70	B	F	6.21	13.9	3	UNK	Schizophrenia
**22**	74	W	F	6.30	7.0	2	ON	Schizophrenia
**23**	81	W	F	5.93	12.5	0.5	OFF	Schizophrenia
**24**	82	W	F	5.89	8.8	1	OFF	Schizophrenia
**25**	86	W	F	5.80	18.2	3	ON	Schizophrenia
**26**	73	W	M	6.15	8.8	3	ON	Schizophrenia
**27**	90	W	F	5.97	7.8	0.5	ON	Schizophrenia
**28**	77	W	M	6.40	24.0	3	OFF	Schizophrenia
**29**	73	W	M	6.35	7.2	0	ON	Schizophrenia
**30**	70	W	M	6.49	14.3	1	ON	Schizophrenia
**31**	68	W	M	6.27	8.9	N/A	ON	Schizophrenia
**32**	86	W	M	6.48	15.4	3	ON	Schizophrenia
**33**	75	W	M	5.85	5.8	3	ON	Schizophrenia
**34**	81	W	F	6.47	15.1	3	OFF	Schizophrenia
**35**	70	W	M	6.36	17.3	3	OFF	Schizophrenia
**36**	75	W	F	6.49	21.5	0.5	UNK	Schizophrenia
**37**	62	W	F	6.74	23.7	0	ON	Schizophrenia
**38**	71	W	M	N/A	9.5	3	ON	Schizophrenia
**39**	83	W	M	N/A	16.3	3	OFF	Schizophrenia
**40**	61	W	M	N/A	6.2	2	ON	Schizophrenia

Supplementary Table 1 (S1). Demographics of anterior cingulate cortex (ACC) subjects from the Mount Sinai NIH Brain and Tissue Repository. Subject identification numbers, age, race, sex, pH, PMI, CDR, medication status, and diagnosis listed for all subjects used in study. *Abbreviations*: *PMI,* postmortem interval (hours); CDR, clinical dementia rating; MED, medication; *UNK*, unknown; *W*, white; *B*, black; *F,* female; *M* male; *N/A*, not available.

ASSAY	GENE NAME	PRIMER
Adenosine 1 Receptor (A1R)	ADORA1	Hs00181231_m1
Adenosine 2A Receptor (A2AR)	ADORA2A	Hs00169123_m1
Cyclophilin A	PPIA	Hs99999904_m1
Beta Actin	ACTB	Hs99999903_m1
Beta2-Microglobulin	B2M	Hs99999907_m1
Glyceraldeyde-3-phosphate dehydrogenase	GAPDH	Hs99999905_m1

Supplementary Table 2 (S2). TaqMan primers. Assay, gene name, and IDs for all primers used in study.

Supplementary Figures & Figure Captions.

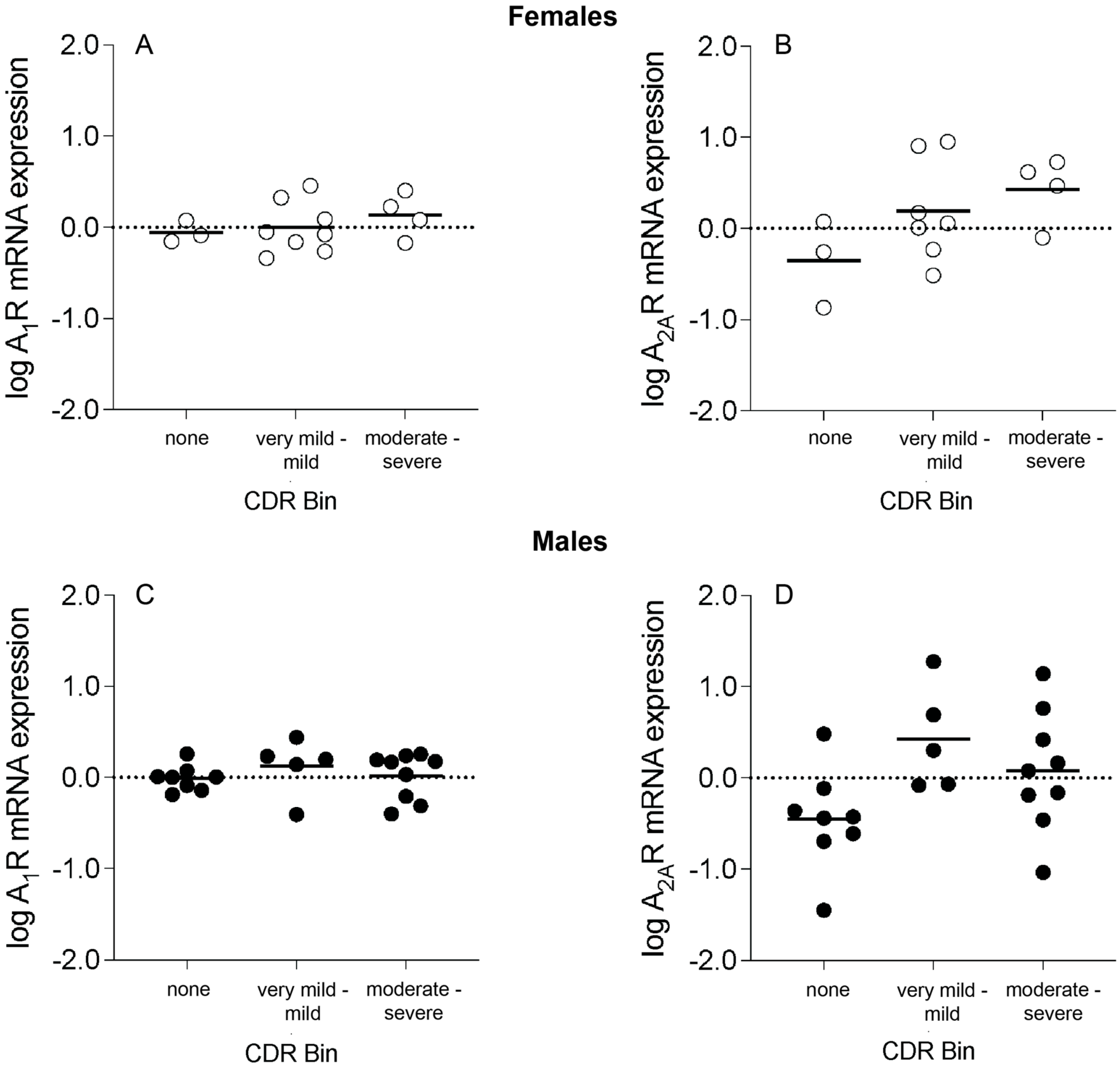

Supplementary Figure 1 (S1). Adenosine A_1 receptor (A_1R) and A_{2A} receptor (A_{2A}R) mRNA expression in an enriched population of anterior cingulate cortex (ACC) pyramidal neurons in control (CTL) and schizophrenia (SCZ) subjects binned according to clinical dementia rating (CDR) score in female and male subjects. A) A_1R mRNA expression (ANOVA: $F_{(2,12)} = 1.038$, $p = 0.384$) and B) A_{2A}R mRNA expression (ANOVA: $F_{(2,11)} = 0.792$, $p = 0.477$) were not significantly different in female subjects across CDR bins. C) A_1R mRNA expression (ANOVA: $F_{(2,19)} = 0.495$, $p = 0.617$) and D) A_{2A}R mRNA expression (ANCOVA: $F_{(2,18)} = 1.219$, $p = 0.319$, after controlling for the effect of PMI) were not significantly different in male subjects across CDR bins. n = 3-9/group. Open circles indicate female subjects and closed circles indicate male subjects. Data presented as mean.

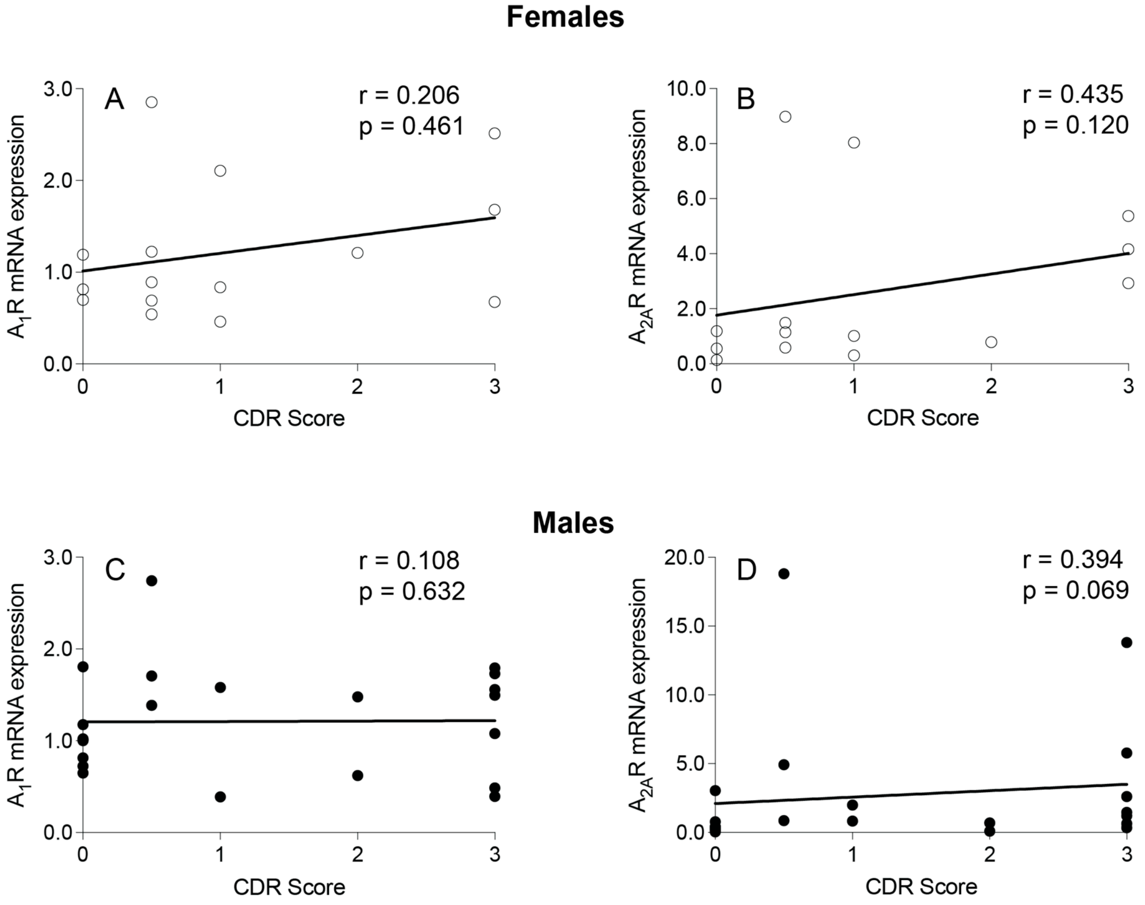

Supplementary Figure 2 (S2). Spearman's correlation analysis between adenosine A_1 receptor (A_1R) and A_{2A} receptor ($A_{2A}R$) mRNA expression and clinical dementia rating (CDR) scores in female and male subjects. A) No significant associations were observed between A_1R mRNA expression or B) $A_{2A}R$ mRNA expression and CDR scores in female subjects. C) No significant associations were observed between A_1R mRNA expression or D) $A_{2A}R$ mRNA expression and CDR scores in male subjects. CDR scores: 0 = no dementia, 0.5-1 = very mild-mild dementia, 2-3 = moderate to severe dementia. n = 14-23/group. Open circles indicate female subjects and closed circles indicate male subjects.

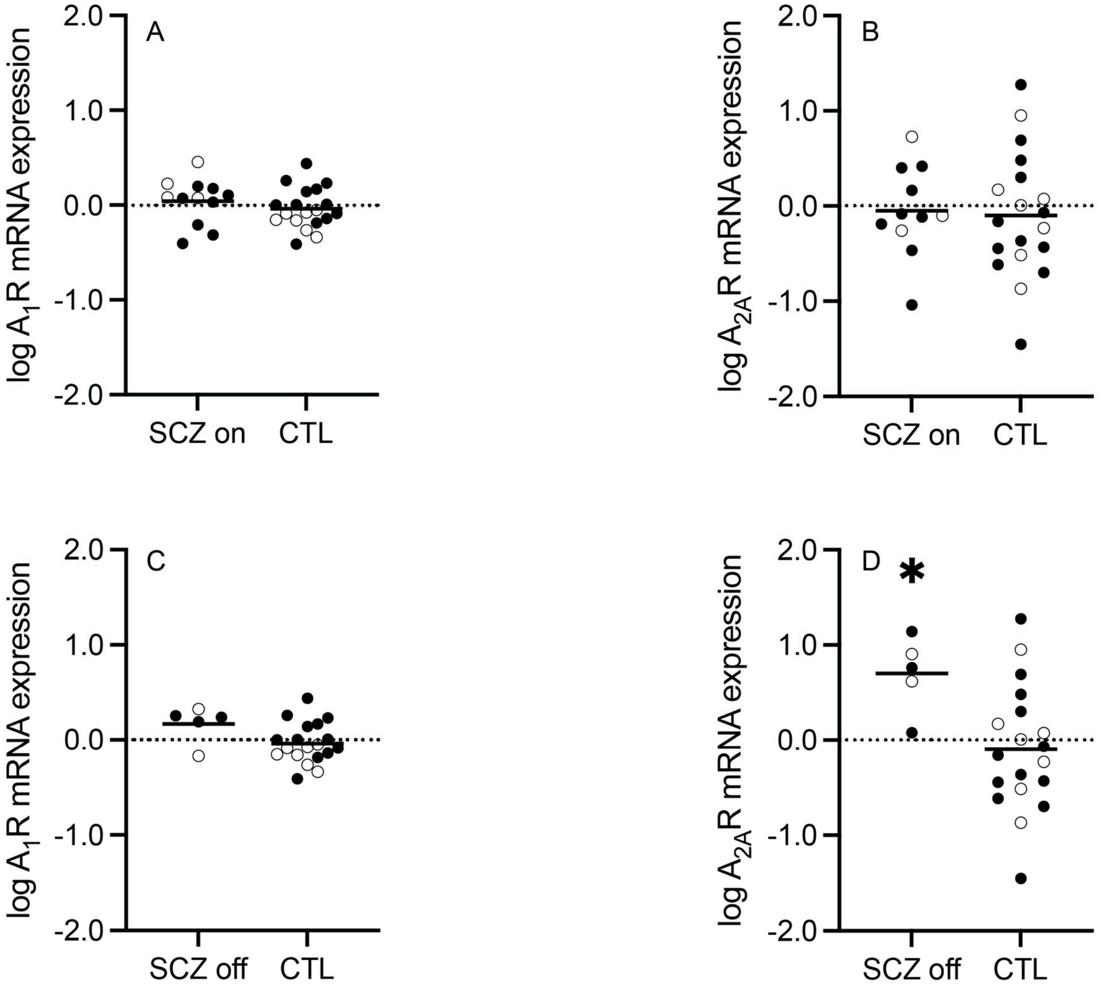

Supplementary Figure 3 (S3). Adenosine A_1 receptor (A_1R) and A_{2A} receptor (A_{2A}R) mRNA expression in an enriched population of anterior cingulate cortex (ACC) pyramidal neurons in schizophrenia (SCZ) subjects on or off antipsychotic medication vs. control (CTL) subjects. A) A_1R mRNA expression ($t_{(29)} = -0.950$, $p = 0.350$) and B) A_{2A}R mRNA expression ($t_{(28)} = -0.224$, $p = 0.825$) were not significantly different between SCZ subjects on antipsychotic medication and control subjects. C) A_1R mRNA expression ($t_{(22)} = -1.943$, $p = 0.065$) was not significantly different between SCZ subjects off antipsychotic medication and control subjects. D) A_{2A}R mRNA expression ($t_{(22)} = -2.580$, $p = 0.017$) was significantly increased between SCZ subjects off antipsychotic medication and control subjects. n = 5-19/group. Open circles indicate female subjects and closed circles indicate male subjects. Data presented as mean. * $p < 0.05$.

Supplementary References.

[1] M. S. Sodhi, M. Simmons, R. McCullumsmith, V. Haroutunian, and J. H. Meador-Woodruff, "Glutamatergic gene expression is specifically reduced in thalamocortical projecting relay neurons in schizophrenia," (in eng), *Biol Psychiatry,* vol. 70, no. 7, pp. 646-54, Oct 01 2011, doi: 10.1016/j.biopsych.2011.02.022.

[2] R. E. McCullumsmith *et al.*, "Cell-specific abnormalities of glutamate transporters in schizophrenia: sick astrocytes and compensating relay neurons?," (in eng), *Mol Psychiatry,* vol. 21, no. 6, pp. 823-30, Jun 2016, doi: 10.1038/mp.2015.148.

[3] S. M. O'Donovan *et al.*, "Cell-subtype-specific changes in adenosine pathways in schizophrenia," (in eng), *Neuropsychopharmacology,* vol. 43, no. 8, pp. 1667-1674, 07 2018, doi: 10.1038/s41386-018-0028-6.
